# Hedgehog signaling enhances the Schwann-like and nerve repair-supportive properties of ectomesenchymal stem cells

**DOI:** 10.3389/fnins.2026.1857431

**Published:** 2026-06-24

**Authors:** Dengxin Zhang, Ruxi Bian, Yaqiong Ma, Huiqing Yuan, Xuechao Wu, Hong Tang, Feifei Zhao

**Affiliations:** 1 Department of Infectious Diseases, Affiliated Children’s Hospital of Jiangnan University (Wuxi Children’s Hospital), Wuxi, Jiangsu, China; 2 Department of Anesthesiology, Affiliated Women’s Hospital of Jiangnan University, Jiangsu, China; 3 Jiangnan University Medical Center, Wuxi, Jiangsu, China

**Keywords:** differentiation, EMSCs, PNI, Schwann cells, Shh

## Abstract

**Introduction:**

Peripheral nerve injury (PNI) is characterized by limited regenerative capacity and incomplete functional recovery. Schwann cells (SCs) are essential for nerve repair, but their clinical application is constrained by limited availability. Ectomesenchymal stem cells (EMSCs), derived from neural crest lineage, represent a promising alternative; however, their inefficient differentiation into SC-like cells remains a key limitation. This study investigated whether activation of Hedgehog signaling via Sonic hedgehog (Shh) could enhance SC-like differentiation and improve nerve regeneration.

**Methods:**

EMSCs were isolated from rat nasal mucosa and transduced with adenoviral vectors to overexpress Shh. SC-like differentiation was assessed using RT-qPCR, Western blot, immunofluorescence, and ELISA. Transcriptomic analysis compared EMSCs with primary SCs. A short-gap rat sciatic nerve defect model was established as an initial proof-of-concept *in vivo* model, and animals received vehicle, EMSCs, Shh-EMSCs, or autograft treatment. Functional recovery, electrophysiology, histology, and ultrastructural analyses were performed.

**Results:**

Transcriptomic analysis revealed that EMSCs possess a partial SC-related transcriptional profile but lack sufficient Hedgehog activation. Shh overexpression activated canonical Hedgehog signaling, evidenced by increased Gli1/2 expression and nuclear translocation. Shh-EMSCs showed enhanced expression of SCs markers (P75, GFAP, MBP, S100β), increased secretion of neurotrophic factors (BDNF, NT-3), and reduced inflammatory cytokines. *In vivo*, Shh-EMSCs significantly improved functional recovery, nerve conduction velocity, and gait performance compared with EMSCs alone. Histological and ultrastructural analyses demonstrated increased axonal regeneration, improved organization, and enhanced myelination compared with unmodified EMSCs, although autograft repair remained superior or more complete in several outcome measures.

**Conclusion:**

Hedgehog signaling contributes to SC-like differentiation of EMSCs. Shh-mediated activation promotes a pro-regenerative phenotype and enhances nerve repair-related outcomes in a short-gap sciatic nerve defect model, suggesting that Shh-EMSCs may serve as a potential cell-based strategy for peripheral nerve repair.

## 1 Introduction

Peripheral nerve injury (PNI) is often associated with incomplete functional recovery, especially when long segmental nerve defects are present. In the peripheral nervous system (PNS), myelin sheaths are formed by Schwann cells (SCs), which provide trophic, structural, and insulating support to axons and enable rapid saltatory nerve conduction (Wu et al., 2024; Zhang N. et al., 2024). Following nerve injury, mature SCs exhibit remarkable phenotypic plasticity. They demyelinate, dedifferentiate, proliferate, and acquire a repair-associated phenotype, thereby creating a growth-supportive environment for Wallerian degeneration, axonal regrowth, and remyelination (Majd et al., 2023; Huang et al., 2024). Thus, SC plasticity is not only a cellular response to injury, but also a key biological mechanism underlying peripheral nerve regeneration (Yao et al., 2024).

For long segmental nerve defects, autologous nerve grafting remains the most frequently used clinical strategy and is still considered the gold standard for PNI repair (Li et al., 2025). The therapeutic benefit of autologous nerve grafts is largely attributed to the presence of viable SCs, preserved extracellular matrix architecture, and native guidance structures, which together provide physical guidance and neurotrophic support for regenerating axons (Rao et al., 2022; Yang et al., 2025). However, autologous nerve transplantation requires the sacrifice of healthy donor nerves and may result in donor-site sensory or motor dysfunction, neuroma formation, and scar formation (Xu et al., 2024). These limitations have encouraged the development of alternative regenerative strategies that can reproduce the supportive functions of autologous nerve grafts while avoiding donor-site morbidity.

Cell-based therapies, particularly SC transplantation, have therefore attracted increasing attention for peripheral nerve repair (Abusalah et al., 2024; Mili and Choudhary, 2024). Because SCs directly participate in axonal guidance, trophic support, and remyelination, supplementation with exogenous SCs represents a biologically rational approach to enhance nerve regeneration. Nevertheless, the direct clinical application of autologous SCs remains challenging. Adult SCs are usually obtained from peripheral nerve biopsies, which may cause additional donor-site injury, and the number of cells that can be harvested is limited (Afjeh-Dana et al., 2022; Crabtree et al., 2024). Although current techniques enable the extraction and purification of autologous SCs, obtaining a sufficient quantity of high-quality cells for effective transplantation remains difficult (Chua et al., 2023). These limitations highlight the need for an alternative, expandable cell source that can acquire SC-like properties without requiring sacrifice of healthy peripheral nerves.

From a developmental perspective, SCs are derived from neural crest cells through Schwann cell precursor intermediates (Stierli and Sommer, 2022). This developmental origin provides a rationale for using neural crest-derived stem cells as candidate sources for generating SC-like cells. Craniofacial tissues comprise both neural crest-derived and non-neural crest-derived cellular components. In the context of the present study, we focus on neural crest-derived cell populations within craniofacial tissues, because they may represent biologically relevant sources for SC-like cell generation (Kastriti et al., 2022; Leathers and Rogers, 2022). As a craniomaxillofacial tissue-derived stem cell population, nasal mucosa-derived ectomesenchymal stem cells (EMSCs) originate from the neural crest (Shi et al., 2023). Previous investigations have identified nasal mucosa-derived EMSCs as neural crest-derived cells that can be readily isolated, exhibit low immunogenicity, possess multilineage and neural differentiation potential, and provide neurotrophic and extracellular matrix support for neural differentiation (Deng et al., 2019; Zhang N. et al., 2024). Together, these properties suggest that EMSCs may represent a practical and biologically relevant seed cell source for generating SC-like cells.

However, the ability of EMSCs to support peripheral nerve repair depends not only on their accessibility and expansion capacity, but also on whether they can be efficiently directed toward a SC-like phenotype. At present, the molecular mechanisms regulating SC-like differentiation of EMSCs remain insufficiently understood, which restricts their application in sciatic nerve regeneration. Hedgehog signaling is closely associated with neural crest lineage regulation and peripheral nerve biology (Bos et al., 2025; Chevalier et al., 2026). Therefore, we hypothesized that activation of Hedgehog signaling by Sonic hedgehog (Shh) may promote the SC-like differentiation of EMSCs and enhance their regenerative capacity after peripheral nerve injury.

Although *in vitro* induction assays are useful for determining whether EMSCs acquire SC-associated molecular features, they cannot fully predict whether these cells can promote nerve repair after transplantation. In the present study, the *in vitro* experiments were designed to evaluate whether Shh activation could enhance SC-like differentiation of EMSCs, whereas a short-gap rat sciatic nerve defect model was used as an initial proof-of-concept *in vivo* model to determine whether this induced phenotype could translate into functional and structural nerve repair-related outcomes. Functional peripheral nerve repair requires coordinated axonal regrowth, SC-mediated remyelination, inflammatory regulation, and target reinnervation, which cannot be fully reproduced by *in vitro* marker analysis alone. Therefore, we used this short-gap rat sciatic nerve defect model to further evaluate the regenerative effects of Shh-EMSCs *in vivo* (Wu et al., 2025; Zhou et al., 2025).

The aim of the present study was to determine whether EMSCs can be directed toward a SC-like phenotype and to clarify whether Hedgehog signaling contributes to this process. We first compared the transcriptomic profiles of EMSCs and primary SCs to identify potential regulatory differences associated with SC-like differentiation. We then activated Hedgehog signaling in EMSCs through Shh overexpression and evaluated SC-associated marker expression, Gli1/2 activation, neurotrophic factor secretion, and inflammatory cytokine production *in vitro*. Finally, we assessed the regenerative effects of Shh-EMSCs in a short-gap rat sciatic nerve defect model as an initial proof-of-concept *in vivo* evaluation using functional, electrophysiological, histological, immunostaining, and ultrastructural analyses. This study provides evidence that Shh-mediated Hedgehog activation enhances the SC-like differentiation and regenerative potential of EMSCs, supporting Shh-EMSCs as a candidate cell-based strategy for peripheral nerve repair.

## 2 Materials and methods

### 2.1 Isolation and culture of EMSCs

Rat EMSCs were isolated from nasal mucosal tissue harvested from SD rats as previously described (Shi et al., 2021). Fresh tissue was rinsed three times in sterile phosphate-buffered saline (PBS) containing 1% penicillin/streptomycin to remove blood and debris. The tissue was mechanically minced into ∼1 mm^3^ fragments using sterile ophthalmic scissors without enzymatic digestion, and the fragments were placed into 6-well plates pre-coated with 0.1% gelatin. Explants were cultured in DMEM/F12 (HyClone) supplemented with 10% FBS (HyClone), 1% GlutaMAX, and 1% penicillin/streptomycin. Cultures were maintained at 37°C in a humidified incubator with 5% CO_2_. After 5–7 days, spindle-shaped cells migrated out from the tissue pieces and adhered to the plate. Tissue remnants were removed, and adherent cells were expanded by replacing medium every 2–3 days. Cells were subcultured with 0.25% trypsin-EDTA when reaching 80–90% confluence. EMSCs at passages 3–5 were used for all subsequent experiments.

### 2.2 Adenoviral transduction of Shh

For Shh overexpression, EMSCs were transduced with a recombinant adenovirus carrying the full-length rat Shh cDNA under the CMV promoter. An empty vector adenovirus was used as a negative control. The adenoviral construct and transduction procedure were performed with technical support from Heyuan Biotechnology. Cells were seeded at 60–70% confluence in serum-free medium and exposed to virus at a multiplicity of infection of 50 for 6 h. The medium was then replaced with complete culture medium containing 10% FBS. After 48 h, cells were harvested to evaluate adenoviral transduction efficiency and Shh overexpression at both the mRNA and protein levels. Shh mRNA expression was measured by qRT-PCR, and Shh protein expression was confirmed by Western blotting. Only cultures showing > 2.5-fold upregulation of Shh mRNA together with detectable elevation of Shh protein relative to vector controls were included in downstream experiments.

All procedures involving recombinant adenoviruses were performed under biosafety level 2 (BSL-2) conditions in accordance with institutional biosafety regulations and the Biosafety in Microbiological and Biomedical Laboratories (BMBL) 6th Edition guidelines (Meechan and Potts, 2020). Viral preparation, cell transduction, and medium replacement were conducted in a certified Class II biosafety cabinet. Adenovirus-contaminated consumables and culture waste were disinfected and disposed of according to institutional biosafety procedures.

### 2.3 CCK-8-based cell viability and growth assessment

Cell viability and metabolic activity-based growth were assessed using the Cell Counting Kit-8 (CCK-8, Dojindo, Japan). Briefly, cells were seeded in 96-well plates at 5 × 10^3^ cells per well in quintuplicate. At each time point, 10 μL of CCK-8 reagent was added to each well and incubated for 2 h at 37°C. Absorbance at 450 nm was measured using a microplate reader (BioTek, United States). Because CCK-8 detects cellular dehydrogenase activity, the absorbance value was interpreted as an indirect indicator of viable cell number and metabolic activity rather than a direct measurement of cell proliferation. Growth curves were plotted based on absorbance values and compared between groups.

### 2.4 Schwann cell-like differentiation induction

To induce Schwann cell-like differentiation, EMSCs and Shh-EMSCs were plated onto laminin-coated culture dishes at a density of 5 × 10^4^ cells/cm^2^. The induction medium consisted of DMEM/F12 supplemented with 10% FBS, 5 μM forskolin (Sigma), 10 ng/mL basic fibroblast growth factor (bFGF, PeproTech), 10 ng/mL platelet-derived growth factor-AA (PDGF-AA, PeproTech), and 50 ng/mL recombinant human neuregulin-1β1 (R&D Systems). The medium was refreshed every 48 h. After 14 days of induction, cells were harvested for subsequent qRT-PCR, Western blotting, immunofluorescence staining, and ELISA analyses. Morphological changes toward an elongated, bipolar, or spindle-like Schwann cell-like appearance were recorded under an optical microscope.

### 2.5 RNA extraction and qRT-PCR

RNA extraction and quantitative RT-PCR were performed as previously described with minor modifications (Lyu et al., 2020). Briefly, total RNA was isolated using TRIzol reagent (Invitrogen, United States) according to the manufacturer’s instructions. RNA concentration and purity were assessed using a NanoDrop 2,000 spectrophotometer. For reverse transcription, 1 μg of RNA was converted into cDNA using the PrimeScript RT reagent kit (Takara, Japan). Quantitative PCR was then carried out using SYBR Premix Ex Taq II (Takara, Japan) on an ABI 7,500 Real-Time PCR System. GAPDH was used as the internal control, and relative gene expression levels were calculated using the 2^∧^−ΔΔCt method. The genes analyzed included Hedgehog pathway-related genes (Shh, Gli1, and Gli2), Schwann cell-associated markers (P75, GFAP, MBP, and S100β), neurotrophic factors (BDNF and NT-3), and inflammatory cytokines (IL-1 and TNFα). Each reaction was performed in triplicate. Primer sequences are listed in Table 1.

**TABLE 1:** Primer sequences used for RT-qPCR analysis.

Gene	Forward primer sequence (5–3)	Reverse primer sequence
Shh	GTAACGCTACGAGA GGAGGC	GAGCACCCGGTTG ATGAGAA
Gli1	AGCCCTGGACCA CGCATC	AATATGTGGGAAAG GCGCGG
Gli2	GGATAGCAGCTTCC CCGAC	GGTGTCTCATGTC AATCGGC
GFAP	AGGCTAATGACTAT CGCCGC	GCCTCTCCAAGGA CTCGTTC
MBP	CTCCGAGGCGTAGAG GAACT	CACCACTGTCCAAT CAGGGC
S100β	AAGTCCACACCCA GTCCTCT	GTGCTTGTCACCCT CTCTCC
BDNF	CCCGGCTTGGAGAA GGAAAC	GGGAACCCGGTCT CATCAAA
NT-3	CGAACTCGAGTCCA CCTTT	TGTAAGATCTTCAG GAAGCCCA
IL-1	GACTTCACCATGGAA CCCGT	GGAGACTGCCCAT TCTCGAC
TNFα	GGAGGGAGAACAG CAACTCC	GCCAGTGTATGAGAGG GACG
P75	TGCACAGACTGAC CACCATC	GCGTACAATGCTCCT GGTCT
GAPDH	GCATCTTCTTGTGC AGTGCC	TACGGCCAAATCCG TTCACA

### 2.6 Western blotting

Western blotting was performed as previously described with minor modifications (Shi et al., 2021). Briefly, total proteins were extracted using RIPA lysis buffer supplemented with protease inhibitors (Beyotime, China) on ice for 30 min. After centrifugation at 12,000 × g for 15 min at 4°C, the supernatants were collected and protein concentrations were measured using a BCA Protein Assay Kit (Thermo Fisher Scientific, United States). Equal amounts of protein (20–30 μg per lane) were denatured at 95°C for 5 min, separated by 10% SDS–PAGE, and transferred onto PVDF membranes (Millipore, United States). The membranes were blocked with 5% non-fat milk in TBST for 1 h at room temperature and incubated overnight at 4°C with primary antibodies against Shh and β-actin. After washing, the membranes were incubated with HRP-conjugated secondary antibodies (1:5,000) for 1 h at room temperature. Protein bands were detected using enhanced chemiluminescence (ECL, Thermo Fisher Scientific, United States), and band intensities were quantified using ImageJ software and normalized to β-actin.

### 2.7 ELISA for secreted factors

Supernatants from EMSCs and Shh-EMSCs after 14 days of induction were collected, centrifuged at 1,500 rpm for 10 min, and stored at −80°C until analysis. Levels of neurotrophic factors (NT-3, BDNF, and Shh) and pro-inflammatory cytokines (TNF-α, IL-1β) were measured using commercial ELISA kits (DUMABIO, China) according to the manufacturer’s instructions. Absorbance was read at 450 nm, and concentrations were determined from standard curves.

### 2.8 Immunofluorescence staining

Cells grown on glass coverslips were fixed with 4% paraformaldehyde for 20 min and permeabilized with 0.1% Triton X-100 for 10 min. After blocking with 5% bovine serum albumin (BSA) for 1 h, cells were incubated overnight at 4°C with primary antibodies against Gli1, Gli2, Sox2, P75, CD44, MBP, and S100β. After washing, Alexa Fluor 488- or 594-conjugated secondary antibodies (1:500, Invitrogen) were applied for 1 h at room temperature. Nuclei were counterstained with DAPI (Sigma) for 5 min. Coverslips were mounted with antifade medium and visualized using a confocal laser scanning microscope (Leica, Germany). For quantification of immunofluorescence staining used for EMSC characterization, marker-positive cells and DAPI-positive nuclei were counted from randomly selected fields using ImageJ software, and the percentage of marker-positive cells was calculated. The quantified data are provided in Supplementary Table 1.

### 2.9 Animal experiments

#### 2.9.1 Experimental animals

Adult male Sprague-Dawley rats (weight 200–250 g, age 8–10 weeks) were obtained from the Sibeifu biotech. Animals were housed under standard conditions (22 ± 2°C, 12 h light/dark cycle) with free access to food and water. All experimental procedures were approved by the Institutional Animal Care and Use Committee of Jiangnan university and performed in accordance with the National Institutes of Health Guide for the Care and Use of Laboratory Animals.

#### 2.9.2 Euthanasia method

Rats were euthanized by inhalation of an overdose of isoflurane. Animals were placed in an induction chamber, and isoflurane was delivered at 5% in 100% oxygen at a flow rate of 1 L/min until respiratory arrest occurred. After confirming the absence of breathing and heartbeat, the animals remained in the chamber for an additional 10 min to ensure death.

#### 2.9.3 Sciatic nerve injury model and cell transplantation

Rats were randomly allocated to four groups before surgery using a random number-based method (*n* = 6 per group): control, EMSCs, Shh-EMSCs, and autograft groups. Only male rats were used in this study to reduce biological variability associated with sex hormone fluctuations and the estrous cycle in this initial proof-of-concept investigation. Rats were anesthetized by intraperitoneal injection of 1% pentobarbital sodium (40 mg/kg). The right sciatic nerve was exposed through a gluteal muscle-splitting incision, and a 5 mm segment was excised at the mid-thigh level to establish a short-gap sciatic nerve defect model. In the control, EMSCs, and Shh-EMSCs groups, a silicone conduit (10 mm in length, inner diameter 1.5 mm) was used to bridge the nerve gap, with both proximal and distal nerve stumps inserted 2.5 mm into the conduit and secured with microsutures. This model was used as an initial proof-of-concept model to compare the regenerative effects of EMSCs and Shh-EMSCs under the same conduit-based repair condition, and was not intended to represent a critical-sized nerve defect model. Matrigel was used as a cell-retention carrier to maintain the transplanted cells within the silicone conduit and at the injury site. To control for potential vehicle effects, all conduit-based groups received the same volume of Matrigel/PBS vehicle. Specifically, the control group received blank Matrigel/PBS vehicle without cells; the EMSCs group received Matrigel/PBS vehicle containing EMSCs at a density of 1 × 10^6^ cells in 20 μL; and the Shh-EMSCs group received the same Matrigel/PBS vehicle containing Shh-EMSCs at the same cell density. Thus, the control, EMSCs, and Shh-EMSCs groups shared the same vehicle background, and the difference among these groups was the presence or type of transplanted cells. In the autograft group, the excised nerve segment was reversed and reimplanted to bridge the defect. Following implantation, muscle and skin were sutured in layers. Rats were allowed to recover under standard housing conditions, and postoperative care included intraperitoneal administration of penicillin (50,000 U/day) for 3 consecutive days to prevent infection. Investigators responsible for postoperative behavioral assessment, CatWalk gait analysis, electrophysiological measurements, histological and immunostaining evaluation, transmission electron microscopy quantification, and statistical analysis were blinded to group allocation until data analysis was completed.

#### 2.9.4 Assessment of sciatic nerve functional index and tibial functional index

Functional recovery of the sciatic nerve was evaluated using walking track analysis to calculate the sciatic functional index (SFI) and tibial functional index (TFI). At designated time points post-surgery, rats were allowed to walk along a confined corridor lined with white paper after their hind paws were dipped in ink. Footprints were collected and analyzed to obtain parameters including print length (PL), toe spread (TS), and intermediary toe spread (IT). SFI and TFI values were calculated according to established formulas described by Bain et al. (1989). A value close to 0 indicates normal nerve function, whereas values approaching -100 represent severe functional impairment.

#### 2.9.5 Gait analysis using the CatWalk system

Automated gait analysis was performed using the CatWalk system (Noldus, Wageningen, Netherlands) preoperatively and at 8 and 12 weeks post-surgery. Rats were placed on a glass walkway (100 × 15 × 0.6 cm) and allowed to walk freely. Fluorescent light emitted from an encased lamp was reflected along the glass plate, illuminating the paw contact areas. Runs were captured by a high-speed video camera positioned underneath the glass plate and analyzed using Catwalk software v10.5 (Noldus). Three compliant runs were recorded per animal at each time point. The following gait parameters were analyzed: maximum contact area (ipsilateral/contralateral ratio), maximum contact intensity (ipsilateral/contralateral ratio), stand time (s), and swing speed (ipsilateral/contralateral ratio). All parameters were quantified and compared among experimental groups.

#### 2.9.6 Nerve conduction velocity measurement

Electrophysiological recovery was assessed by direct sciatic nerve conduction velocity (NCV) measurement at defined postoperative time points, rather than by somatosensory evoked potential (SEP) or motor evoked potential (MEP) testing. Briefly, the sciatic nerve was re-exposed under anesthesia, and bipolar electrodes were placed at the proximal and distal sites of the regenerated nerve segment for electrical stimulation and recording. NCV was calculated from the distance between the two electrode sites and the latency difference of the evoked responses.

#### 2.9.7 Histological and immunohistochemical analysis

Regenerated nerve grafts were harvested at 12 weeks. Samples were fixed in 4% paraformaldehyde, dehydrated, embedded in paraffin, and sectioned at 5 μm. Sections were stained with HE and toluidine blue to evaluate morphology and myelination. For immunohistochemistry, sections were incubated with primary antibodies against S100β and MBP overnight at 4°C, followed by HRP-conjugated secondary antibodies. Positive staining was visualized using 3,3′-diaminobenzidine (DAB) and counterstained with hematoxylin.

#### 2.9.8 Transmission electron microscopy

A portion of regenerated nerve tissue was fixed in 2.5% glutaraldehyde overnight, post-fixed in 1% osmium tetroxide, dehydrated in graded ethanol, and embedded in epoxy resin. Ultrathin sections (70 nm) were stained with uranyl acetate and lead citrate, and examined under a transmission electron microscope (JEOL, Japan) to observe axonal regeneration and myelin sheath formation. For TEM-based quantification, regenerated nerve tissues from three animals per group were analyzed. Myelin sheath thickness was measured from multiple myelinated axons using ImageJ. The mean myelin sheath thickness was first calculated for each animal, and the animal-level mean value was then used for intergroup statistical analysis. Thus, the animal, rather than the individual myelinated fiber, was considered the statistical unit.

#### 2.9.9 Ethics approval and consent to participate

This study did not involve human participants or human tissue samples. All experimental procedures were conducted using rat-derived tissues and were approved by the Institutional Animal Care and Use Committee of Jiangnan University (Approval No.: JN.No20231115S0800530[542]). All methods were performed in accordance with the relevant institutional guidelines and regulations.

### 2.10 Statistical analysis

Data are presented as mean ± standard deviation (SD). Statistical analyses were performed using GraphPad Prism 9. Comparisons between two groups were performed using unpaired Student’s *t*-test. For comparisons among multiple groups at a single time point, one-way ANOVA followed by Tukey’s *post hoc* multiple-comparison test was used. For longitudinal functional assessments measured repeatedly in the same animals, including SFI, TFI, CatWalk gait parameters, and NCV, two-way repeated-measures ANOVA was performed with treatment group and time as the two factors, followed by *post hoc* multiple-comparison tests. When missing values were present, a mixed-effects model was used instead of repeated-measures ANOVA. A value of *p* < 0.05 was considered statistically significant.

## 3 Results

### 3.1 Isolation and identification of nasal mucosal EMSCs

**FIGURE 1:**
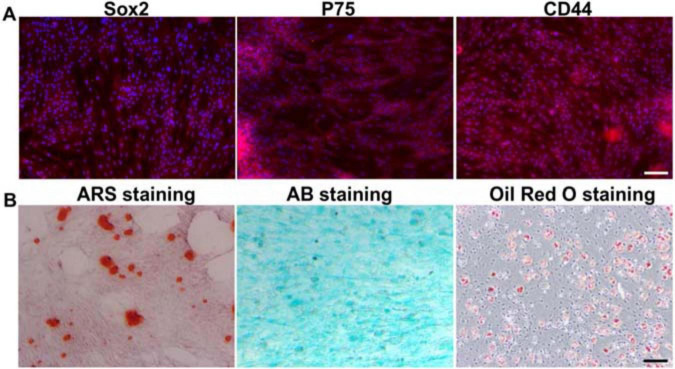
Characterization of ectomesenchymal stem cells. **(A)** Representative immunofluorescence staining images showing positive expression of neural crest-associated and mesenchymal markers, including Sox2, P75, and CD44, in ectomesenchymal stem cells. Nuclei were counterstained with DAPI. Quantification of marker-positive cells is provided in Supplementary Table 1. **(B)** Multilineage differentiation of ectomesenchymal stem cells. Osteogenic differentiation was evaluated by Alizarin Red S (ARS) staining, showing mineralized nodule formation; chondrogenic differentiation was assessed by Alcian blue (AB) staining, indicating glycosaminoglycan deposition; and adipogenic differentiation was demonstrated by Oil Red O staining, showing intracellular lipid droplet accumulation. Scale bars = 50 μm.

Under an optical microscope, EMSCs displayed a typical spindle-shaped or fusiform morphology with a relatively uniform cytoplasmic appearance. These morphological features were based on qualitative observation rather than quantitative measurement. Immunofluorescence staining further confirmed the stem cell phenotype of EMSCs, as the cells positively expressed neural crest-associated and stemness markers, including SOX2, p75 (NGFR), and CD44 (Figure 1A). Quantitative analysis of marker-positive cells showed that a high proportion of EMSCs expressed these markers, further supporting their neural crest-associated stem cell phenotype (Supplementary Table 1). Representative optical microscopic images of P3 and P5 EMSCs further showed that cells maintained a typical spindle-shaped or fusiform morphology during short-term passaging (Supplementary Figure S1).

To evaluate their multilineage differentiation potential, P4 EMSCs were subjected to adipogenic, osteogenic, and chondrogenic induction (Figure 1B). Following osteogenic induction, abundant mineralized nodules were observed by Alizarin Red staining, indicating robust osteogenic capacity. Chondrogenic differentiation resulted in the formation of highly aggregated cell masses that stained positively with Alcian Blue. After adipogenic induction, intracellular lipid droplets were detected by Oil Red O staining, demonstrating adipogenic differentiation capability. Collectively, these results indicate that EMSCs exhibit characteristic morphology, express neural crest-associated stem cell markers, and possess robust multilineage differentiation potential, consistent with our previous findings.

### 3.2 Bulk RNA-seq-based transcriptomic comparison between EMSCs and primary SCs

To investigate the molecular basis underlying the limited SC-like differentiation potential of EMSCs, we performed bulk RNA sequencing and compared the transcriptomic profiles of EMSCs with those of primary SCs. Bulk RNA-seq analysis revealed a clear divergence in signaling state, with primary SCs showing selective activation of Hedgehog signaling and EMSCs retaining a mixed, partially accessible SC/myelin-related repertoire. Consistently, volcano plot analysis demonstrated widespread transcriptional differences between EMSCs and SCs, with a total of 2,959 significantly differentially expressed genes (DEGs), including 760 upregulated and 2,199 downregulated genes (Supplementary Figure S2). The distribution of DEGs revealed a predominance of downregulated genes in EMSCs relative to SCs, indicating that a substantial portion of the SCs transcriptional program is not fully activated in EMSCs. Notably, the Hedgehog ligand Desert hedgehog (Dhh) was robustly enriched in SCs (FPKM≈3.03 in SCs vs. ≈ 0.00 in EMSCs), accompanied by upregulation of the canonical effector Gli1 (logFC ≈ + 6.94), indicating an active Dhh signaling pathway in SCs. By contrast, EMSCs already expressed several SC/myelin markers at baseline, including Pmp22 (≈121.54 vs. 21.44), Mpz/P0 (≈0.61 vs. 0.04), Mbp (≈0.24 vs. 0.00), Egr2/Krox20 (≈3.22 vs. 0.67), Ncam1 (≈16.24 vs. 4.56), S100b (≈8.86 vs. 0.05), and Adgrg6/Gpr126 (≈3.00 vs. 0.19), despite overall transcriptomic distance from SCs (all FDR < 0.05 where applicable). SCs also displayed higher Gfap (≈0.69 vs. 0.07; logFC ≈ + 2.99), consistent with a glial/repair-skewed state, and Erbb3 was elevated in SCs (≈1.10 vs. 0.03; logFC ≈ + 4.76) (Supplementary Table S1). Together, these data support a working model in which EMSCs retain a permissive SCs-related substrate but lack robust upstream Hedgehog input; the SC-enriched activation of Dhh and Gli1 suggests that Dhh/Hedgehog signaling may represent a candidate upstream pathway contributing to the acquisition of a SC-like phenotype by EMSCs.

To functionally assess whether Hedgehog signaling contributes to SC-like differentiation, EMSCs were treated with exogenous Sonic hedgehog (Shh). qPCR analysis demonstrated robust activation of the Hedgehog pathway, as evidenced by significant upregulation of canonical target genes, including Gli1, Ptch, and Smo. Importantly, Shh stimulation also markedly increased the expression of SCs–associated markers, including P75, GFAP, MBP, and S100β (Supplementary Figure S3).

Collectively, these findings support a model in which EMSCs possess a partial Schwann-related transcriptional profile but lack sufficient upstream Hedgehog activation. Exogenous stimulation of Hedgehog signaling is sufficient to enhance both pathway activity and SC-like gene expression, suggesting that Hedgehog signaling functions as a key upstream driver facilitating the transition of EMSCs toward a SCs-like phenotype.

### 3.3 Shh overexpression enhances the SC-like phenotype of EMSCs

**FIGURE 2:**
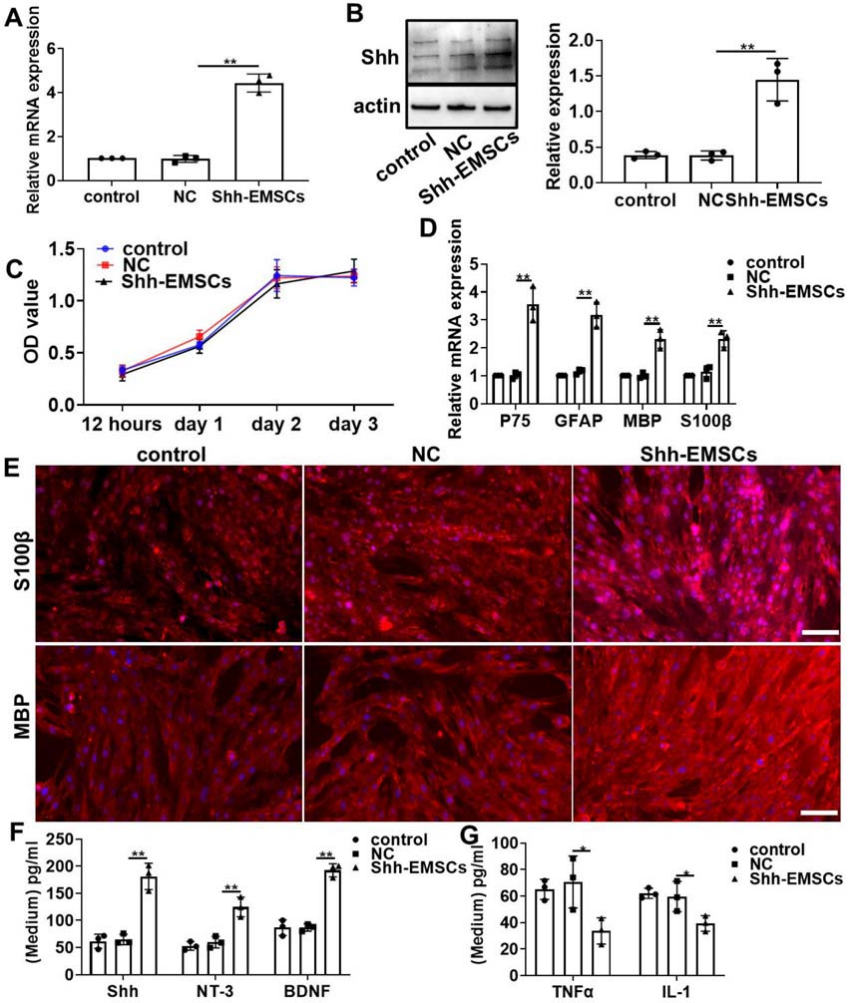
Shh overexpression enhances SC-like differentiation and neurotrophic phenotype of ectomesenchymal stem cells. **(A)** RT-qPCR analysis of Shh expression in EMSCs following adenoviral transduction, showing a significant increase in Shh mRNA levels in Shh-EMSCs compared with vector controls. **(B)** Western blot analysis confirming elevated Shh protein expression in Shh-EMSCs. **(C)** CCK-8 assay showing comparable metabolic activity-based growth profiles between Shh-EMSCs and control EMSCs, indicating that Shh overexpression did not markedly impair short-term cell viability or growth. **(D)** RT-qPCR analysis of SC–associated markers (P75, GFAP, MBP, and S100β) after 14 days of induction, demonstrating significantly enhanced expression in Shh-EMSCs. **(E)** Representative immunofluorescence staining images showing the staining pattern and cellular distribution of MBP and S100β after Schwann cell-like induction. Nuclei were counterstained with DAPI. **(F,G)** ELISA analysis showing increased secretion of neurotrophic factors (Shh, NT-3, and BDNF) and decreased levels of pro-inflammatory cytokines (TNF-α and IL-1) in Shh-EMSCs compared with controls. Data are presented as mean ± SD (*n* = 3). Statistical significance was determined using unpaired Student’s *t*-test. *P < 0.05, **P < 0.01. Scale bars = 50 μm.

To further examine whether Hedgehog activation enhances SC-like differentiation, we generated EMSCs with adenoviral Shh overexpression. (Shh-EMSCs). Transduction efficiency was verified by both RT-qPCR and Western blot analysis. RT-qPCR showed that Shh mRNA expression increased by approximately 4.5-fold compared with vector controls, and Western blot confirmed a marked elevation of Shh protein levels (Figures 2A,B). CCK-8 assays showed comparable metabolic activity-based growth profiles between Shh-EMSCs and vector-control EMSCs, suggesting that Shh overexpression did not markedly impair cell viability or short-term cell growth under the present experimental conditions (Figure 2C). Following 14 days of SCs induction, Shh-EMSCs exhibited significantly enhanced expression of SC/myelin markers, including P75, GFAP, MBP, and S100β, compared with induced control EMSCs (Figure 2D). Representative immunofluorescence staining was performed to visualize the protein-level staining pattern and cellular distribution of MBP and S100β after Schwann cell-like induction. Consistent with the qRT-PCR quantification of SC-associated markers shown in Figure 2D, MBP and S100β staining appeared stronger in Shh-EMSCs than in control EMSCs (Figure 2E). At the secretory level, ELISA analysis revealed increased production of neurotrophic factors, including Shh, NT-3, and BDNF, along with reduced levels of pro-inflammatory cytokines TNF-α and IL-1 in Shh-EMSCs compared with controls (Figures 2F,G). Collectively, these results demonstrate that Shh overexpression enhances SC-like differentiation of EMSCs *in vitro* without markedly impairing short-term cell viability or growth, while promoting a neurotrophic, low-inflammatory phenotype.

### 3.4 Shh activates Hedgehog signaling via Gli1/2 in EMSCs

**FIGURE 3:**
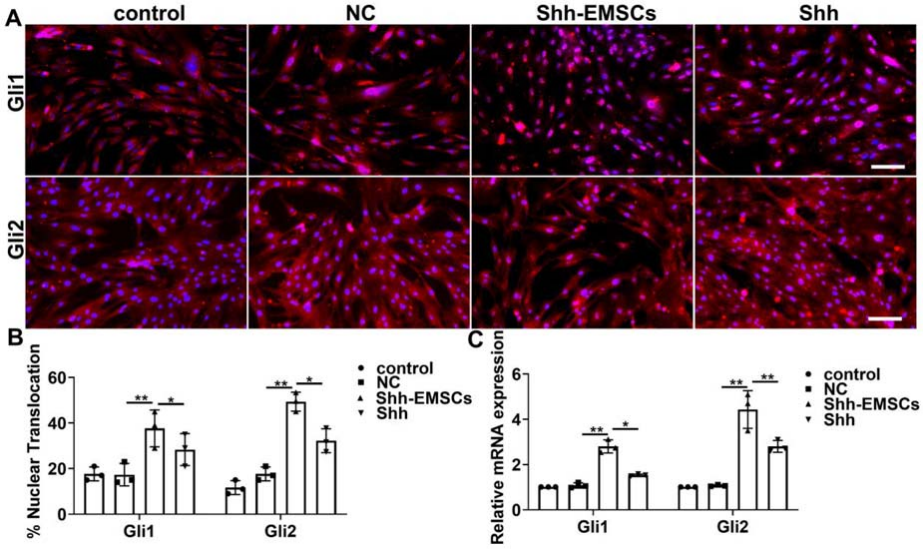
Shh overexpression induces nuclear translocation and transcriptional activation of Gli factors in ectomesenchymal stem cells. **(A)** Immunofluorescence staining of Gli1 and Gli2 showing increased nuclear localization in Shh-EMSCs compared with control EMSCs. Nuclei were counterstained with DAPI. **(B)** Quantification of Gli1 and Gli2 nuclear translocation, showing a significantly higher proportion of cells with nuclear localization in Shh-EMSCs. **(C)** RT-qPCR analysis of Hedgehog pathway activation, demonstrating increased expression of Gli1 and Gli2 in Shh-EMSCs compared with controls and EMSCs treated with exogenous Shh protein. Data are presented as mean ± SD (*n* = 3). Statistical significance was determined using one-way ANOVA followed by *post hoc* multiple comparison test. *P < 0.05, **P < 0.01. Scale bars = 50 μm.

To verify the activation of Hedgehog signaling in Shh-EMSCs, we first examined the subcellular localization of Gli transcription factors by immunofluorescence staining. Both Gli1 and Gli2 exhibited markedly increased nuclear accumulation in Shh-EMSCs compared with control cells, indicating enhanced transcriptional activation of the Hedgehog pathway (Figure 3A). Quantitative analysis of nuclear translocation further confirmed a significant increase in the proportion of cells displaying nuclear localization of Gli1 and Gli2 in Shh-EMSCs, supporting robust pathway activation at the cellular level (Figure 3B). Consistently, RT-qPCR analysis demonstrated significant upregulation of Gli1 and Gli2 transcripts in Shh-EMSCs relative to controls. Notably, the activation level of Hedgehog signaling in Shh-EMSCs was higher than that observed in EMSCs treated with exogenous Shh protein (Figure 3C). Collectively, these results demonstrate that Shh overexpression effectively activates canonical Hedgehog signaling in EMSCs, as evidenced by enhanced nuclear translocation and transcriptional upregulation of Gli1 and Gli2.

### 3.5 Functional recovery assessed by sciatic functional index and tibial functional index

**FIGURE 4:**
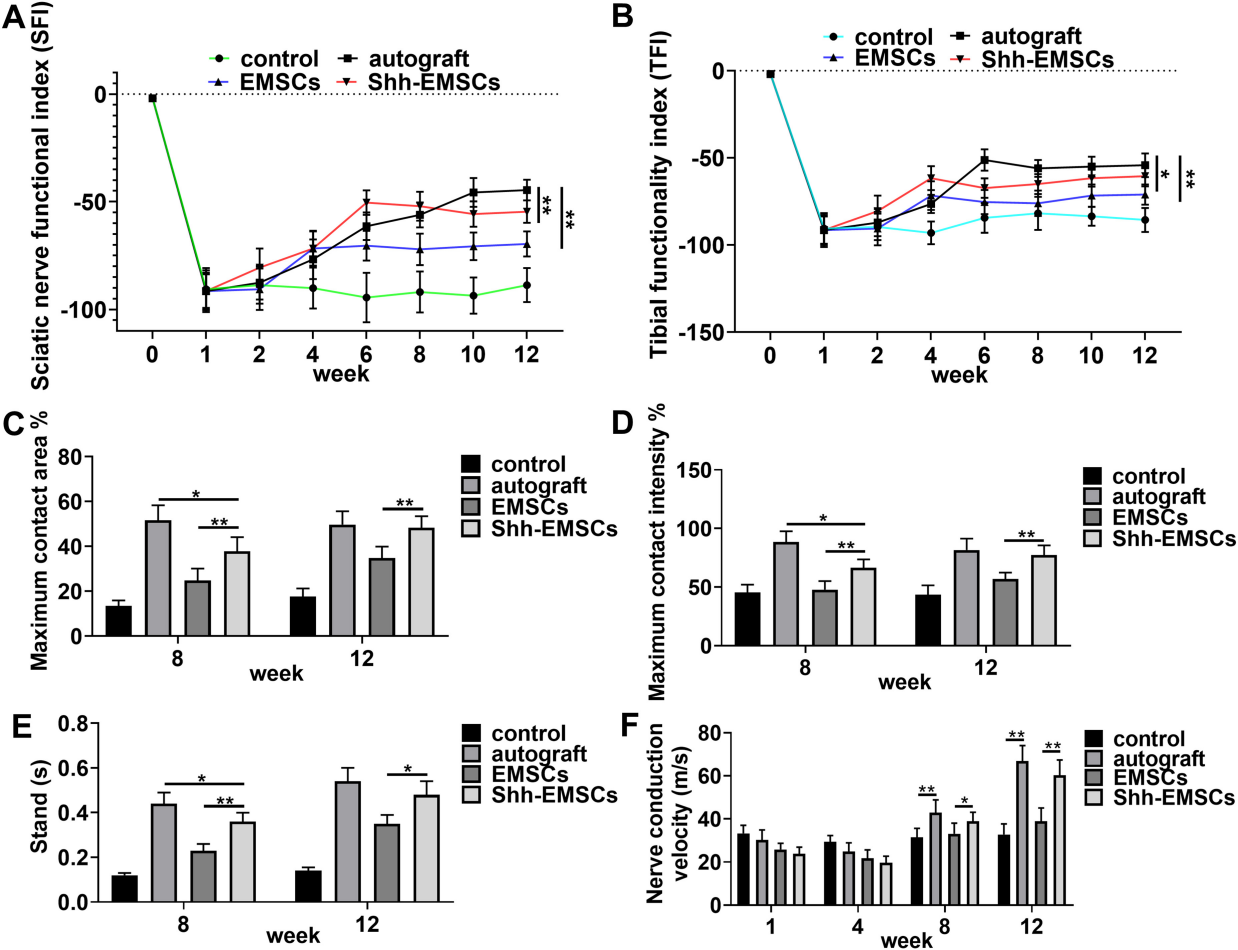
Shh-EMSCs promote functional and electrophysiological recovery in a short-gap rat sciatic nerve defect model. **(A,B)** Functional recovery was evaluated weekly using the sciatic functional index (SFI) and tibial functional index (TFI) over 12 weeks. Longitudinal SFI and TFI data were analyzed using two-way repeated-measures ANOVA followed by *post hoc* multiple-comparison tests. At week 12, the autograft group exhibited the best functional recovery, with SFI and TFI values closest to the pre-injury baseline, while Shh-EMSCs showed significantly improved recovery compared with EMSCs. **(C–E)** Gait analysis was performed using the CatWalk system at weeks 8 and 12. Maximum contact area, contact intensity, and stand time were analyzed using repeated-measures analysis with treatment group and time as factors, followed by *post hoc* multiple-comparison tests. By week 12, Shh-EMSCs showed marked improvement in contact intensity and stand time compared with EMSCs and were not statistically different from autograft for these specific gait parameters. **(F)** Direct sciatic nerve conduction velocity (NCV) was measured at weeks 1, 4, 8, and 12 and analyzed using repeated-measures analysis followed by *post hoc* multiple-comparison tests. At week 12, autograft showed the highest NCV, followed by Shh-EMSCs, EMSCs, and control groups. Data are presented as mean ± SD (*n* = 6 animals per group). *P < 0.05, **P < 0.01.

Functional recovery was evaluated weekly for 12 weeks post-surgery using the SFI and TFI in all experimental groups. Because these parameters were repeatedly measured in the same animals over time, longitudinal data were analyzed using two-way repeated-measures ANOVA followed by *post hoc* multiple-comparison tests. Preoperative SFI and TFI values were near zero in all groups, indicating normal nerve function. Following nerve injury, all groups exhibited severe functional impairment, with SFI (Figure 4A) and TFI (Figure 4B) values approaching −100 at week 1 post-surgery. From week 4 onward, gradual functional recovery was observed in all groups, with intergroup differences becoming more evident after week 8. At week 12 post-surgery, the Autograft group demonstrated the most favorable functional recovery, with SFI and TFI values closest to the pre-injury baseline, as supported by the updated *post hoc* comparisons. The Shh-EMSCs group showed significantly better functional recovery than the EMSCs group, suggesting that activation of Shh signaling enhanced the regenerative capacity of EMSCs under the present short-gap repair condition. The EMSCs group also showed improved functional recovery compared with the conduit-only control group, indicating that EMSCs transplantation alone may support functional recovery in this model. The control group consistently showed the poorest functional recovery throughout the 12-week observation period.

### 3.6 Functional and electrophysiological recovery following nerve regeneration

Gait parameters were analyzed using the CatWalk automated system at weeks 8 and 12 post-surgery to evaluate functional recovery. For maximum contact area (Figure 4C), the autograft group exhibited the highest values at week 8. By week 12, no significant differences were observed among the autograft, Shh-EMSCs, and EMSCs groups for maximum contact area, while all three treatment groups showed significantly greater values than the control group. Both the Shh-EMSCs and EMSCs groups showed significantly greater contact area than the control group at both time points. For maximum contact intensity (Figure 4D), the autograft group consistently displayed the highest values at both weeks 8 and 12. Notably, by week 12, maximum contact intensity in the Shh-EMSCs group was markedly improved compared with the EMSCs group and was not statistically different from the autograft group for this specific parameter, whereas the EMSCs group remained significantly lower. The control group consistently exhibited the lowest values. A similar trend was observed for stand time. At week 8, no significant differences were detected among the autograft, Shh-EMSCs, and EMSCs groups, although all three were significantly improved compared with the control group. By week 12, the autograft and Shh-EMSCs groups demonstrated comparable stand time, both significantly higher than the EMSCs and control groups, while the control group remained the lowest throughout (Figure 4E).

To further assess electrophysiological recovery, nerve conduction velocity (NCV) was measured at weeks 1, 4, 8, and 12 post-surgery (Figure 4F). At week 1 and 4, no significant differences were observed among the autograft, Shh-EMSCs, and EMSCs groups, although all exhibited higher NCV values than the control group. By week 12, the autograft group showed the greatest recovery, followed by the Shh-EMSCs group, which demonstrated substantial improvement but remained lower than autograft. The EMSCs group exhibited only modest recovery, while the control group consistently displayed minimal improvement.

Collectively, these results indicate that Shh-EMSCs enhanced functional and electrophysiological recovery compared with unmodified EMSCs under the present short-gap sciatic nerve defect condition. However, autograft repair remained the most effective treatment in several outcome measures, particularly nerve conduction velocity.

### 3.7 Shh-EMSCs enhanced remyelination and neural regeneration *in vivo*

**FIGURE 5:**
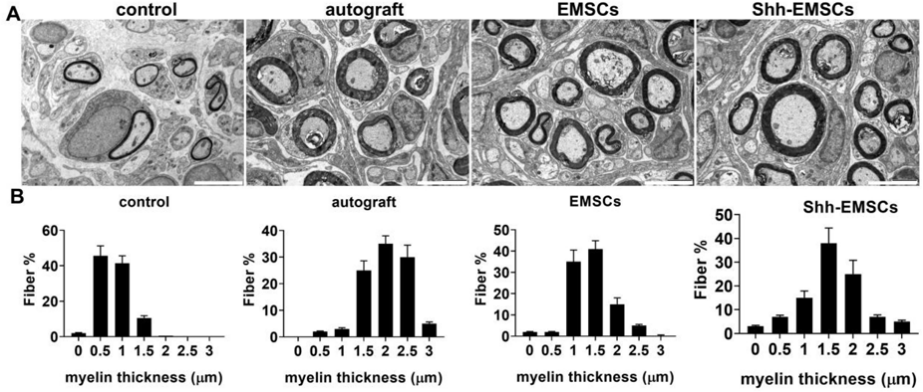
Shh-EMSCs enhance remyelination-related ultrastructural recovery of regenerated nerve fibers. **(A)** Representative TEM images showing regenerated sciatic nerve fibers in each group. autograft, Shh-EMSCs, and EMSCs groups exhibited abundant myelinated axons with relatively organized ultrastructure, whereas the control group showed sparse and disorganized fibers. Scale bars = 4 μm. **(B)** Frequency distribution of myelin sheath thickness in regenerated nerve fibers from each group. For TEM analysis, regenerated nerve samples from three animals per group were analyzed. The corresponding animal-level quantitative comparison of mean myelin sheath thickness and Tukey’s *post hoc* multiple-comparison results are provided in Supplementary Figure S4.

At 12 weeks post-surgery, regenerated sciatic nerves were harvested and evaluated. TEM was performed to assess myelin sheath thickness and the structural characteristics of regenerated nerve fibers, given the critical role of SC-mediated myelination in peripheral nerve regeneration. TEM images revealed regenerated nerve fibers with different degrees of myelination among the groups (Figure 5A). The autograft, Shh-EMSCs, and EMSCs groups showed more abundant myelinated axons with relatively organized ultrastructural features, whereas the control group exhibited sparse and disorganized nerve structures with limited myelination.

Frequency distribution analysis of myelin sheath thickness further showed that the control group contained a higher proportion of thinly myelinated fibers, whereas the autograft and Shh-EMSCs groups displayed a shift toward thicker myelin sheaths (Figure 5B). Animal-level quantification of mean myelin sheath thickness further confirmed the intergroup differences observed in the distribution analysis, and the corresponding statistical comparison is provided in Supplementary Figure S4. The autograft group showed the highest mean myelin sheath thickness, whereas the control group showed the lowest value. Importantly, Shh-EMSCs significantly increased mean myelin sheath thickness compared with unmodified EMSCs, indicating improved remyelination-related ultrastructural outcomes under this short-gap repair condition. These ultrastructural improvements were observed in myelination-related parameters and should not be interpreted as evidence of overall equivalence to autograft repair.

### 3.8 Shh-EMSCs promote structural nerve regeneration and a pro-regenerative microenvironment

**FIGURE 6:**
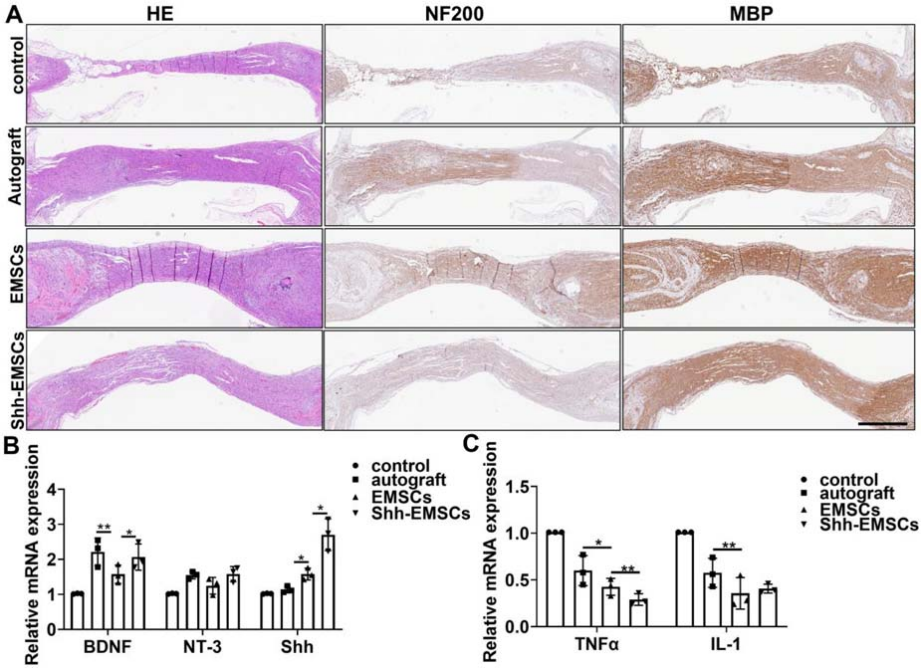
Shh-EMSCs improve structural regeneration-related features and modulate the local microenvironment in a short-gap rat sciatic nerve defect model. **(A)** H&E and immunofluorescence staining (NF200, MBP) showing improved nerve fiber organization and increased axonal and SC-markers in Shh-EMSCs and autograft groups compared with EMSCs and control. **(B,C)** RT-qPCR analysis at 4 weeks showing increased Shh and BDNF expression and reduced TNF-α and IL-1β in Shh-EMSCs compared with control. NT-3 showed no significant difference among groups. Data are presented as mean ± SD (*n* = 3). *P < 0.05, **P < 0.01. Scale bars = 100 μm.

H&E staining showed that regenerated nerve fibers in the Shh-EMSCs and autograft groups exhibited larger diameters and more uniform organization compared with the EMSCs and control groups. Immunofluorescence staining further confirmed enhanced nerve regeneration. NF200 staining revealed a higher density and more continuous distribution of regenerated axons in the Shh-EMSCs and autograft groups, whereas axonal regeneration was less prominent in the EMSCs group and minimal in the control group. MBP staining, a marker of SCs and myelination, showed abundant and evenly distributed MBP-positive structures in both the Shh-EMSCs and autograft groups. In contrast, the EMSCs group displayed fewer MBP-positive fibers, and the control group exhibited only sparse staining (Figure 6A).

To investigate the molecular mechanisms underlying functional recovery, RT-qPCR was performed to evaluate the expression levels of neurotrophic factors (BDNF, NT-3, and Shh) (Figure 6B) and inflammatory cytokines (TNF-α and IL-1β) (Figure 6C) in regenerated sciatic nerve tissues at 4 weeks post-surgery. Shh mRNA expression was significantly upregulated in the Shh-EMSCs group compared to all other groups, confirming successful Shh overexpression in EMSCs prior to transplantation. The autograft and EMSCs groups showed moderate Shh expression levels, while the control group exhibited the lowest Shh expression. BDNF mRNA expression was markedly increased in the Shh-EMSCs group and was not statistically different from that in the autograft group in this assay, with both groups showing significantly higher BDNF levels than the EMSCs and control groups. The EMSCs group exhibited moderate BDNF expression, which was significantly higher than that of the control group. Notably, NT-3 mRNA expression showed no significant differences among all four experimental groups at 4 weeks post-surgery, suggesting that NT-3 may not be a key mediator in the differential regenerative outcomes observed in this study.

Analysis of pro-inflammatory cytokines revealed distinct patterns among the experimental groups. TNF-α and IL-1β mRNA expression were both lowest in the Shh-EMSCs group, indicating a potent anti-inflammatory effect following transplantation of Shh-EMSCs. The EMSCs group also exhibited relatively low expression levels of both cytokines, significantly lower than those in the autograft and control groups. Interestingly, the autograft group showed the highest expression levels of TNF-α and IL-1β among all groups except control, with values significantly exceeding those of the Shh-EMSCs and EMSCs groups. The control group demonstrated the highest inflammatory cytokine expression overall, consistent with the absence of therapeutic intervention and poor regenerative outcome.

These results demonstrate that transplantation of Shh-EMSCs maintained high BDNF expression and significantly suppressed the inflammatory response, as evidenced by the lowest TNF-α and IL-1β levels among all treatment groups. However, these molecular findings should be interpreted as parameter-specific changes rather than evidence of overall equivalence to autograft repair. The elevated Shh expression in the Shh-EMSCs group confirms successful activation of the Shh signaling pathway, which may contribute to the enhanced regenerative phenotype observed in this group.

## 4 Discussion

In the present study, we demonstrated that nasal mucosa-derived EMSCs possess a partial capacity for SC-like differentiation and that activation of Hedgehog signaling through Shh overexpression significantly enhances this process. Transcriptomic comparison between EMSCs and primary SCs showed that although EMSCs already expressed part of a SC-associated transcriptional repertoire, they lacked sufficient upstream Hedgehog pathway activation. On this basis, we further confirmed that Shh stimulation and Shh overexpression both promoted the expression of SC-related markers and activated canonical Hedgehog signaling, as evidenced by increased Gli1/2 expression and nuclear translocation. These findings support the view that Hedgehog signaling is an important regulatory pathway facilitating the transition of EMSCs toward a SC-like phenotype.

Mechanistically, our data suggest that the promotive effect of Shh on SC-like differentiation is closely associated with activation of Gli-dependent transcriptional programs. In Shh-EMSCs, both Gli1 and Gli2 showed enhanced nuclear accumulation, indicating effective activation of downstream Hedgehog signaling. Compared with exogenous Shh protein treatment, adenoviral Shh overexpression produced a stronger activation effect, suggesting that sustained cell-intrinsic pathway activation may be more effective than transient extracellular stimulation in promoting lineage conversion. This observation is important because it indicates that the extent and duration of Hedgehog signaling may influence the differentiation efficiency of EMSCs toward a SC-like state. Nevertheless, because pharmacological or genetic inhibition of Hedgehog signaling was not performed in the present study, our data do not definitively establish that Hedgehog signaling is required for this phenotype. Future studies using Smoothened inhibitors, such as cyclopamine or vismodegib, or direct Gli inhibition will be necessary to confirm pathway specificity and further clarify the downstream mechanisms by which Shh promotes SC-like differentiation of EMSCs.

In addition to molecular changes, Shh activation also improved the functional phenotype of EMSCs *in vitro*. After SC induction, Shh-EMSCs exhibited higher expression of canonical SC-associated markers, including P75, GFAP, MBP, and S100β, together with increased secretion of neurotrophic factors such as BDNF and NT-3. At the same time, the production of pro-inflammatory cytokines was reduced. These findings indicate that Hedgehog activation not only promotes SC-like differentiation at the marker level, but also shifts EMSCs toward a more supportive and regeneration-associated phenotype. Such changes are likely relevant to the *in vivo* setting, where both trophic support and local inflammatory modulation contribute to peripheral nerve repair. The *in vivo* findings further support this interpretation. In the short-gap rat sciatic nerve defect model, transplantation of Shh-EMSCs resulted in significantly improved functional recovery, electrophysiological restoration, and structural regeneration compared with unmodified EMSCs. SFI, TFI, gait parameters, and nerve conduction velocity all showed better recovery trends in the Shh-EMSCs group. Histological, immunostaining, and ultrastructural analyses consistently showed more organized regenerated nerve tissue, increased axonal regeneration, and improved remyelination. Although autograft repair still showed the strongest overall effect, particularly in electrophysiological recovery, Shh-EMSCs achieved substantially better outcomes than EMSCs alone. These findings indicate that targeted activation of Hedgehog signaling can enhance the regenerative performance of EMSCs in peripheral nerve repair, but the effects of Shh-EMSCs should not be interpreted as fully equivalent to autograft repair.

Our findings are consistent with previous studies demonstrating critical roles of Hedgehog signaling in peripheral nerve development and repair (Wu et al., 2022; Xu and Fan, 2025). However, most prior work has focused on embryonic or neonatal SC-precursors, whereas our study extends these observations to neural crest–derived stem cells (Endo et al., 2022; Colchado et al., 2025). We show that EMSCs, which are developmentally related to the neural crest lineage, can be effectively reprogramed toward a SC-like phenotype through Hedgehog activation, thereby supporting their utility as a neural crest–derived cell source for nerve repair.

The use of EMSCs offers several practical advantages over conventional SC transplantation. Autologous SCs are limited by donor-site morbidity, low yield, and restricted expansion capacity (Iacoponi et al., 2023; Han et al., 2025), whereas EMSCs represent an expandable neural crest-derived cell source that can be isolated from nasal mucosal tissue. In addition, EMSCs exhibit low immunogenicity and secrete a range of neurotrophic and extracellular matrix factors that may support nerve regeneration. Given their neural crest origin, EMSCs may also retain a degree of developmental permissiveness toward glial differentiation. In the present study, this possibility was supported by both transcriptomic and functional findings, as EMSCs already displayed partial SC-related molecular features before induction, and these features became more pronounced after Hedgehog activation. Together, these observations suggest that Shh signaling may reinforce a pre-existing but incomplete SC-associated program in EMSCs, thereby improving their suitability for peripheral nerve repair.

Nevertheless, several limitations should be acknowledged. Our results suggest that Hedgehog signaling is associated with EMSC-to-SC-like conversion; however, the downstream transcriptional targets of Gli1/2 and their interactions with other regulatory pathways remain to be clarified. Given that EMSCs and primary SCs represent distinct cell types and differentiation states, transcriptomic enrichment analysis identified multiple biological processes rather than a single dominant mechanism. Therefore, Hedgehog signaling should be considered an important candidate regulatory axis, but not the only pathway involved in SC-like differentiation of EMSCs. Shh-EMSCs showed increased expression of SC-associated markers, enhanced neurotrophic factor secretion, reduced inflammatory cytokine production, and improved regenerative outcomes *in vivo*. However, direct *in vitro* functional assays were not performed in the present study. In particular, dorsal root ganglion neuron co-culture, neuron-glia interaction assays, and *in vitro* myelination assays would provide stronger evidence for functional SC-like properties. Thus, Shh-EMSCs should be described as acquiring a SC-like phenotype rather than being considered fully equivalent to primary SCs. Another limitation is the use of a 5-mm rat sciatic nerve defect model, which does not represent a critical-sized nerve gap. Spontaneous regeneration may occur in this short-gap model, especially when a conduit is used. Therefore, the *in vivo* findings should be interpreted as initial proof-of-concept evidence showing that Shh activation enhances the regenerative performance of EMSCs under the same conduit-based repair condition. Future studies using 10–15 mm critical-sized defect models are needed to validate the efficacy of Shh-EMSCs in more challenging long-gap nerve injuries. Despite the absence of an obvious effect of Shh overexpression on short-term cell viability, the long-term stability, off-target effects, and potential safety concerns associated with sustained Hedgehog activation require further investigation. In addition, only male rats were used in this initial study, and future studies including both sexes will be needed to evaluate potential sex-specific differences.

## 5 Conclusion

This study identifies Hedgehog signaling, particularly Shh-driven Gli activation, as an important mechanism associated with SC-like differentiation of EMSCs. Activation of Hedgehog signaling promoted the acquisition of a SC-like phenotype in EMSCs and enhanced their nerve repair-related effects in a short-gap rat sciatic nerve defect model. These findings support the potential of Shh-EMSCs as a candidate cell-based strategy for peripheral nerve regeneration. Further studies are needed to clarify the downstream molecular mechanisms, perform direct *in vitro* functional assays, track the *in vivo* fate of transplanted cells, evaluate long-term safety, and validate therapeutic efficacy in critical-sized nerve defect models.

## Data Availability

The raw sequence data reported in this paper have been deposited in the Genome Sequence Archive (Genomics, Proteomics & Bioinformatics 2025) in National Genomics Data Center (Nucleic Acids Res 2026), China National Center for Bioinformation/Beijing Institute of Genomics, Chinese Academy of Sciences (GSA: CRA041876; PRJCA062798) that are publicly accessible at https://ngdc.cncb.ac.cn/gsa.
