# Supplementary material for: Hedgehog signaling enhances the Schwann-like and nerve repair-supportive properties of ectomesenchymal stem cells
Source: Front Neurosci. 2026 Jun 24;20:1857431. doi: 10.3389/fnins.2026.1857431 (PMC13341592; doi:10.3389/fnins.2026.1857431)
Supplement: Supplementary file 1 [file Data_Sheet_1.docx]

Supplementary table 1. Quantification of immunofluorescence staining for Sox2, p75, and CD44 in EMSCs.

| Marker | Positive cells (%) |
| --- | --- |
| Sox2 | 97.3 ± 6.7 |
| p75 | 95.6 ± 7.2 |
| CD44 | 94.8 ± 8.1 |

Table 1. Data are presented as mean ± SD. Marker-positive cells were normalized to DAPI-positive nuclei from randomly selected fields.


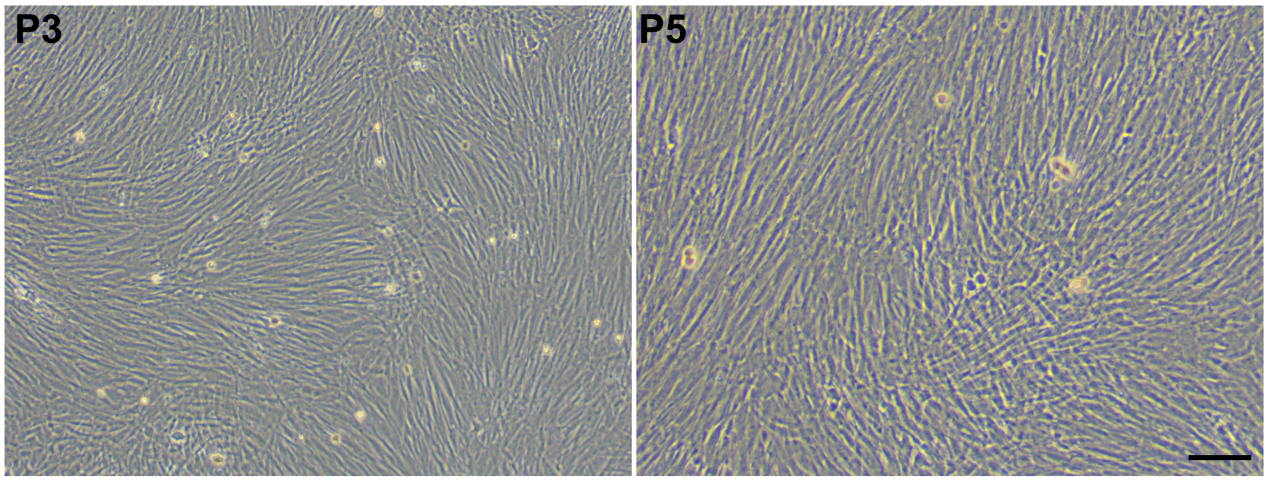


**Figure. S1 Optical microscopic morphology of EMSCs at passages 3 and 5.**

**Representative phase-contrast/optical microscopic images showing that EMSCs maintained a typical spindle-shaped or fusiform morphology from P3 to P5, with no obvious morphological deterioration during short-term passaging. Scale bars = 50 μm.**


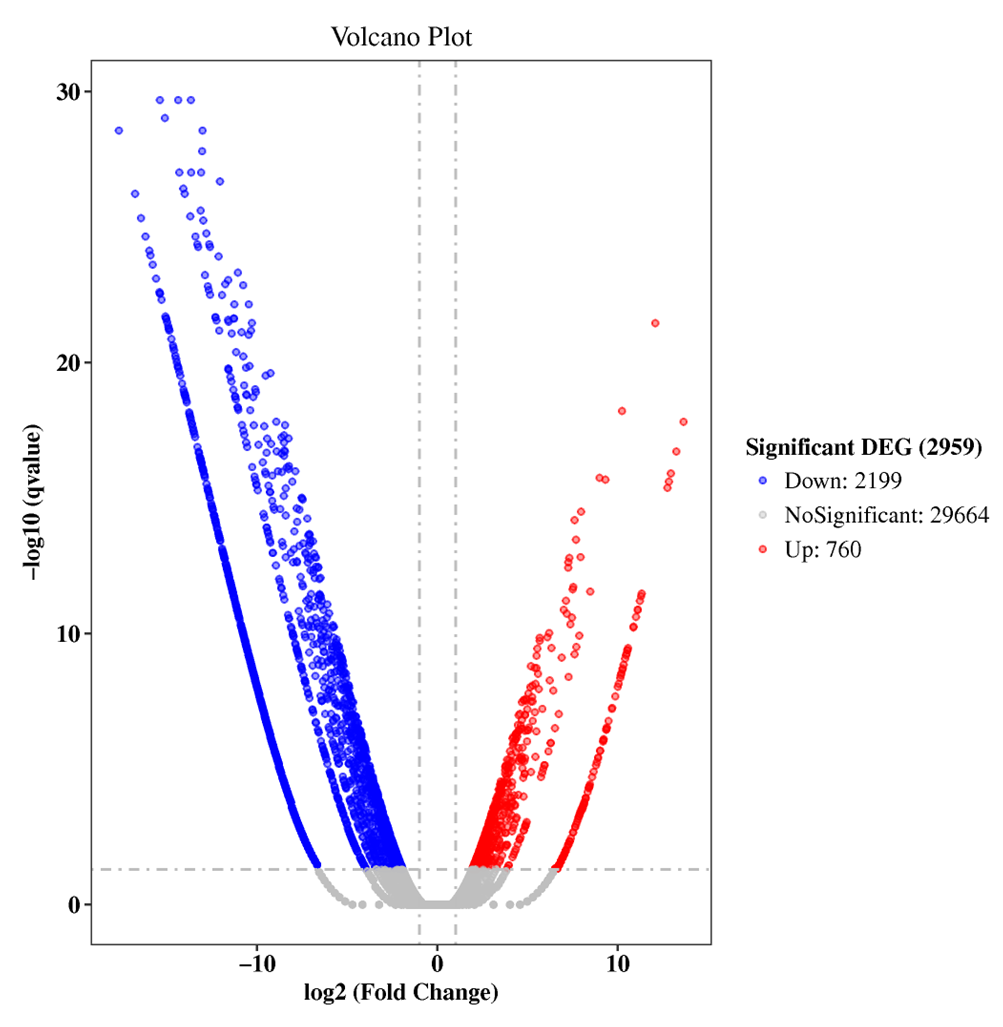


**Figure. S2 Differential gene expression analysis between ectomesenchymal stem cells and primary Schwann cells.** Volcano plot showing differentially expressed genes (DEGs) between EMSCs and SCs. A total of 2,959 DEGs were identified (|log₂FC| > 1, FDR < 0.05), including 760 upregulated and 2,199 downregulated genes in EMSCs relative to SCs. Each dot represents an individual gene, with significantly upregulated genes shown in red and downregulated genes in blue.


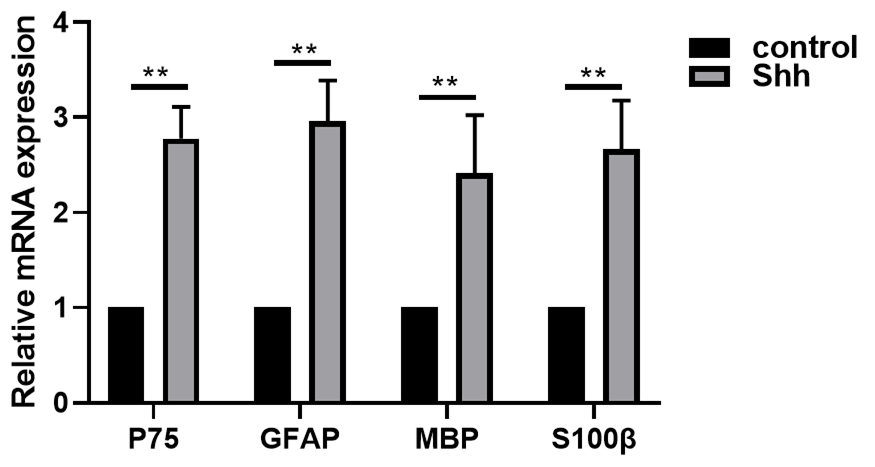


Figure. S3 Hedgehog activation enhances Schwann-like gene expression in ectomesenchymal stem cells. RT-qPCR analysis of Schwann cell–associated markers (P75, GFAP, MBP, and S100β) in ectomesenchymal stem cells following Shh treatment. Expression levels of all markers were significantly increased compared with untreated controls, indicating enhanced Schwann-like differentiation. Data are presented as mean ± SD (n = 3). Statistical significance was determined using unpaired Student’s t-test. **P < 0.01.


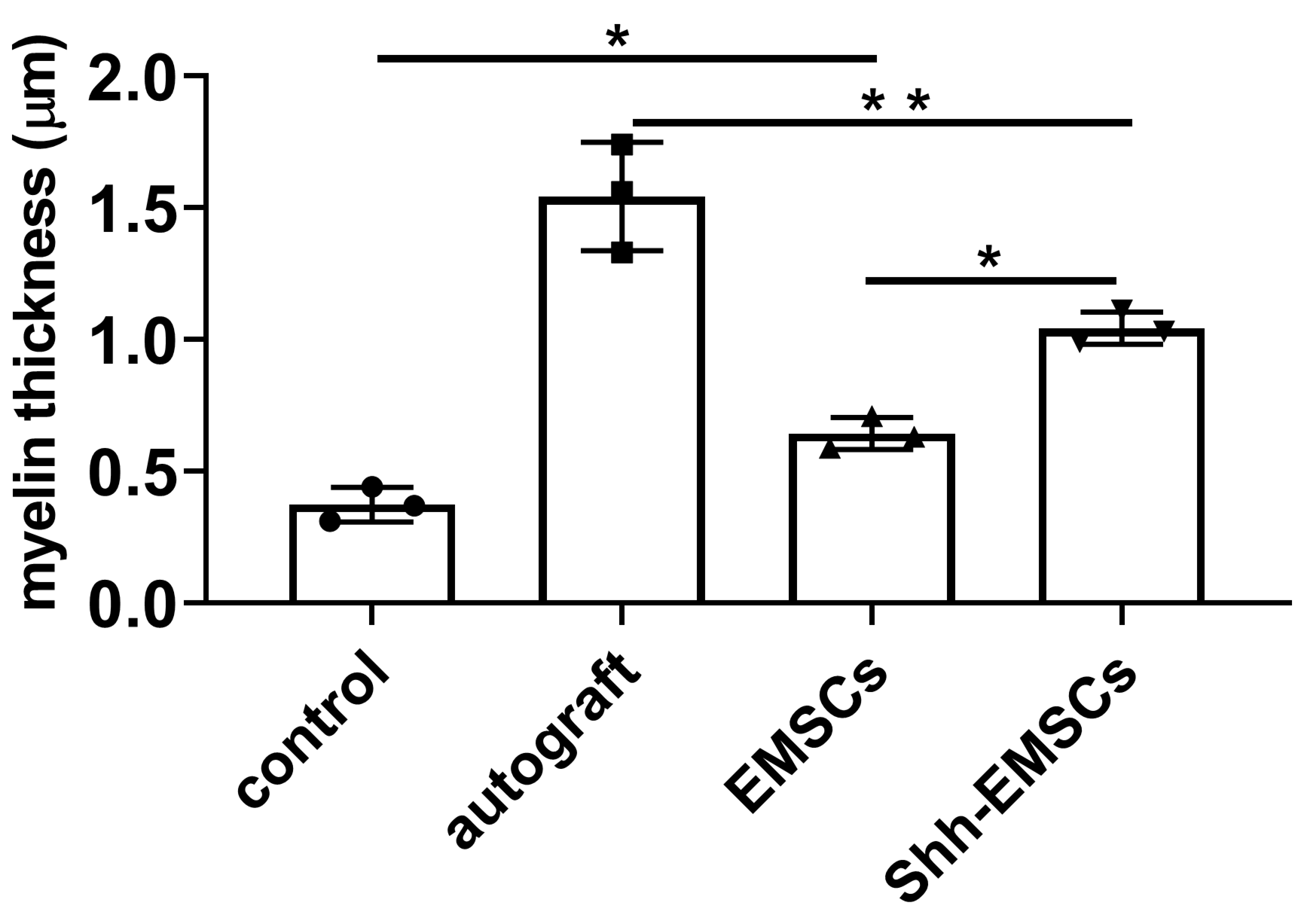


Figure. S4 Quantitative analysis of mean myelin sheath thickness based on TEM images. For TEM-based quantification, regenerated nerve samples from three animals per group were analyzed. Myelin sheath thickness was measured from multiple myelinated axons in each animal, and the animal-level mean value was used for statistical analysis. Data are presented as mean ± SD (n = 3 animals per group). Statistical significance was determined using one-way ANOVA followed by Tukey’s post hoc multiple-comparison test. Post hoc comparison results are indicated in the graph. *P < 0.05, **P < 0.01.
